# Supplementary material for: Human and Bacterial Toll-Interleukin Receptor Domains Exhibit Distinct Dynamic Features and Functions
Source: Molecules. 2022 Jul 14;27(14):4494. doi: 10.3390/molecules27144494 (PMC9318647; doi:10.3390/molecules27144494)
Supplement: Supplementary file 1 [file molecules-27-04494-s001.zip › molecules-1775015-supplementary.pdf]

molecules

MDPI

Supplementary Material

Supplementary Figures

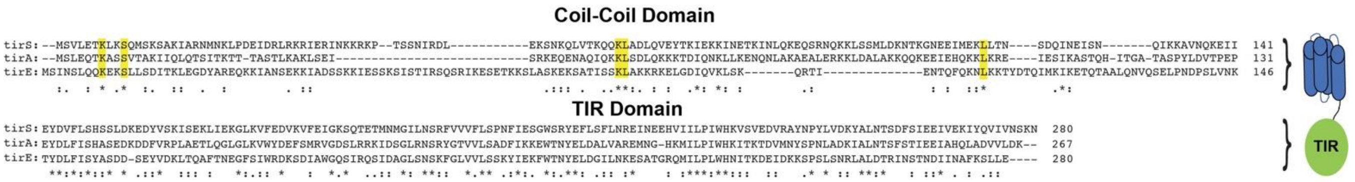

Figure S1. Sequence comparison between tirS, tirA, and tirE. The sequences of all three bacterial TIR-containing proteins used here include tirS from *Staphylococcus aureus*, NCBI reference sequence WP_000114516.1, tirA from *Acinetobacter junii*, GenBank reference sequence RTE47094.1, and tirE from both Enterococcus and an Enterococcus phage, NCBI references sequences YP_009103093.1 and WP_102970086.1, respectively. The N-terminal regions that comprise the CC dimerization domain and the C-terminal regions that comprise the TIR regions are shown along with a schematic illustrating these two domains.

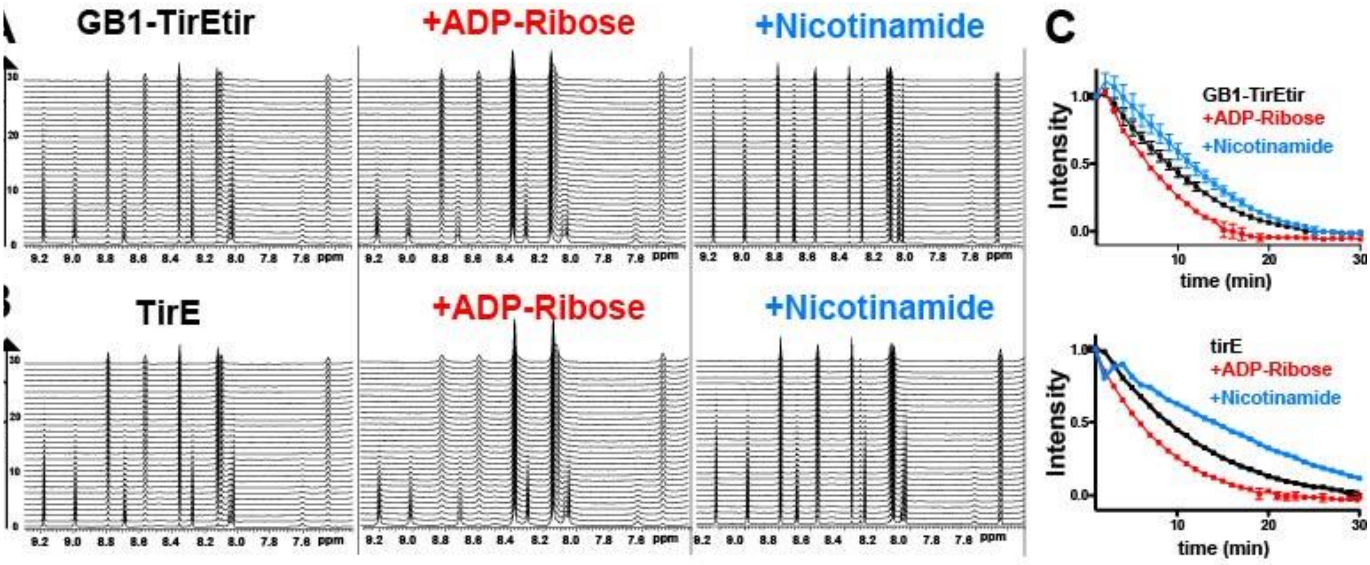

Figure S2. The effects of products on catalysis of tirE. A) 1D NMR spectra following NAD^+ cleavage mediated by 10 μM GB1-tirE TIR alone (left), preincubated with 1 mM ADP-Ribose (center), or preincubated with 1 mM nicotinamide (right). B) Similar 1D NMR spectra are shown with 12.5 μM full-length tirE. C) Normalized intensities as a function of time are shown for both the GB1-tirE TIR construct and full-length tirE. Assays collected with no product are shown (black), with ADP-ribose (red), and with nicotinamide (blue).

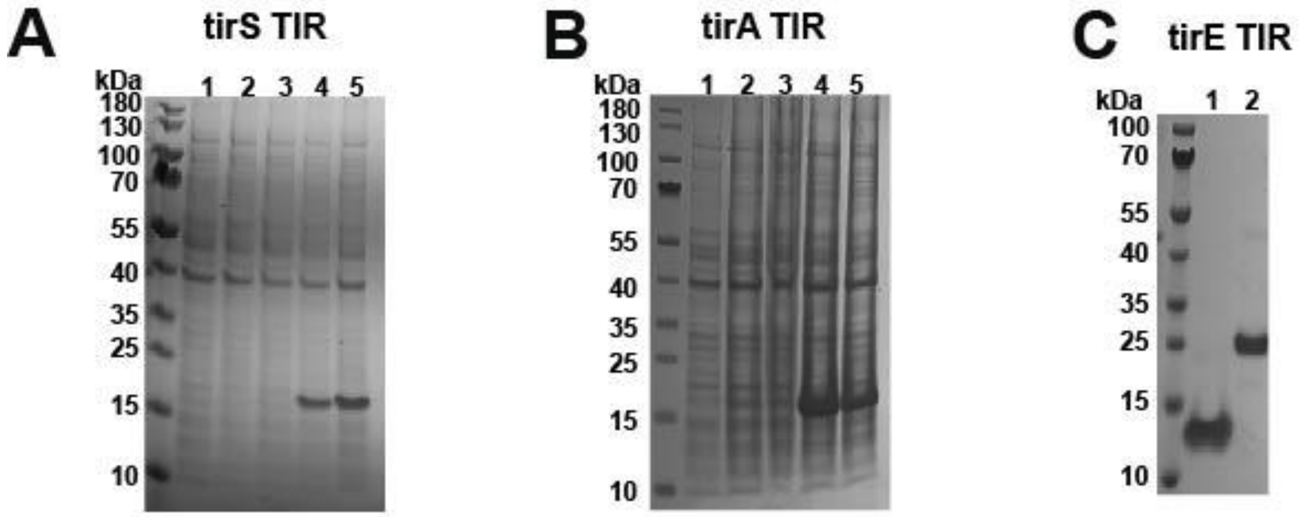

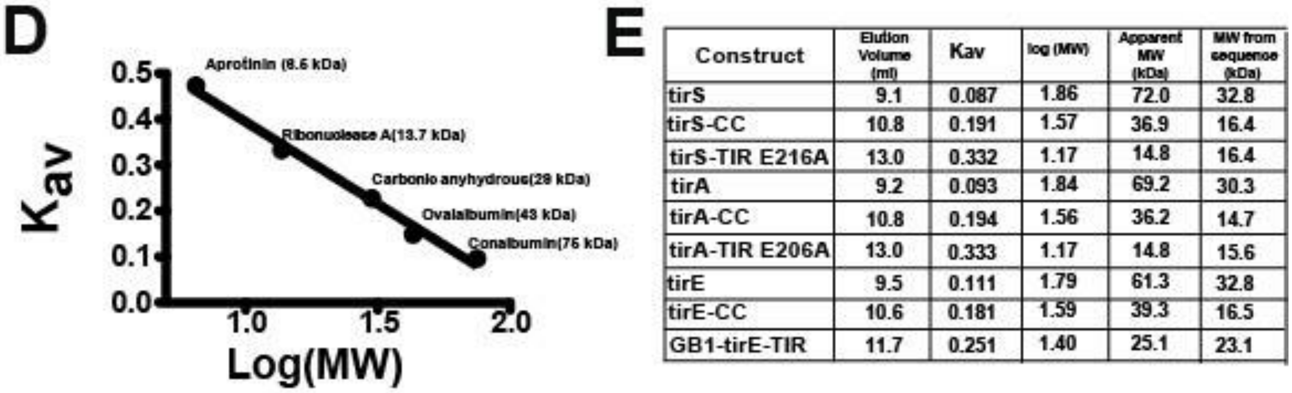

E

Construct	Elution Volume (ml)	Kav	log (MW)	Apparent MW (kDa)	MW from sequence (kDa)
tirS	9.1	0.087	1.86	72.0	32.8
tirS-CC	10.8	0.191	1.57	36.9	16.4
tirS-TIR E216A	13.0	0.332	1.17	14.8	16.4
tirA	9.2	0.093	1.84	69.2	30.3
tirA-CC	10.8	0.194	1.56	36.2	14.7
tirA-TIR E206A	13.0	0.333	1.17	14.8	15.6
tirE	9.5	0.111	1.79	61.3	32.8
tirE-CC	10.6	0.181	1.59	39.3	16.5
GB1-tirE-TIR	11.7	0.251	1.40	25.1	23.1

Figure S3. Biochemical characterization of tirS, tirA, and tirE. A) Recombinant expression of the tirS-TIR domain (residues 141-280) and its E216A mutant. Shown is the pre-induced (lane 1), WT induced in both pLysS (lane 2) and BL21 (lane 3), and E216A mutant in pLysS (lane 4) and BL21 (lane 5). B) Analogous expressions are shown for the tirA-TIR domain (residues 132-267 and mutant E206A). C) Analytical Superdex-75 standard curve. D) Analytical Superdex-75 analysis of all tirS, tirA, and tirE constructs assessed here based on the standard curve using. Kav was calculated as the difference between the Elution Volume and void volume (7.73 ml determined from dextran blue) divided by the difference between the column volume (23.5 ml) and void volume.

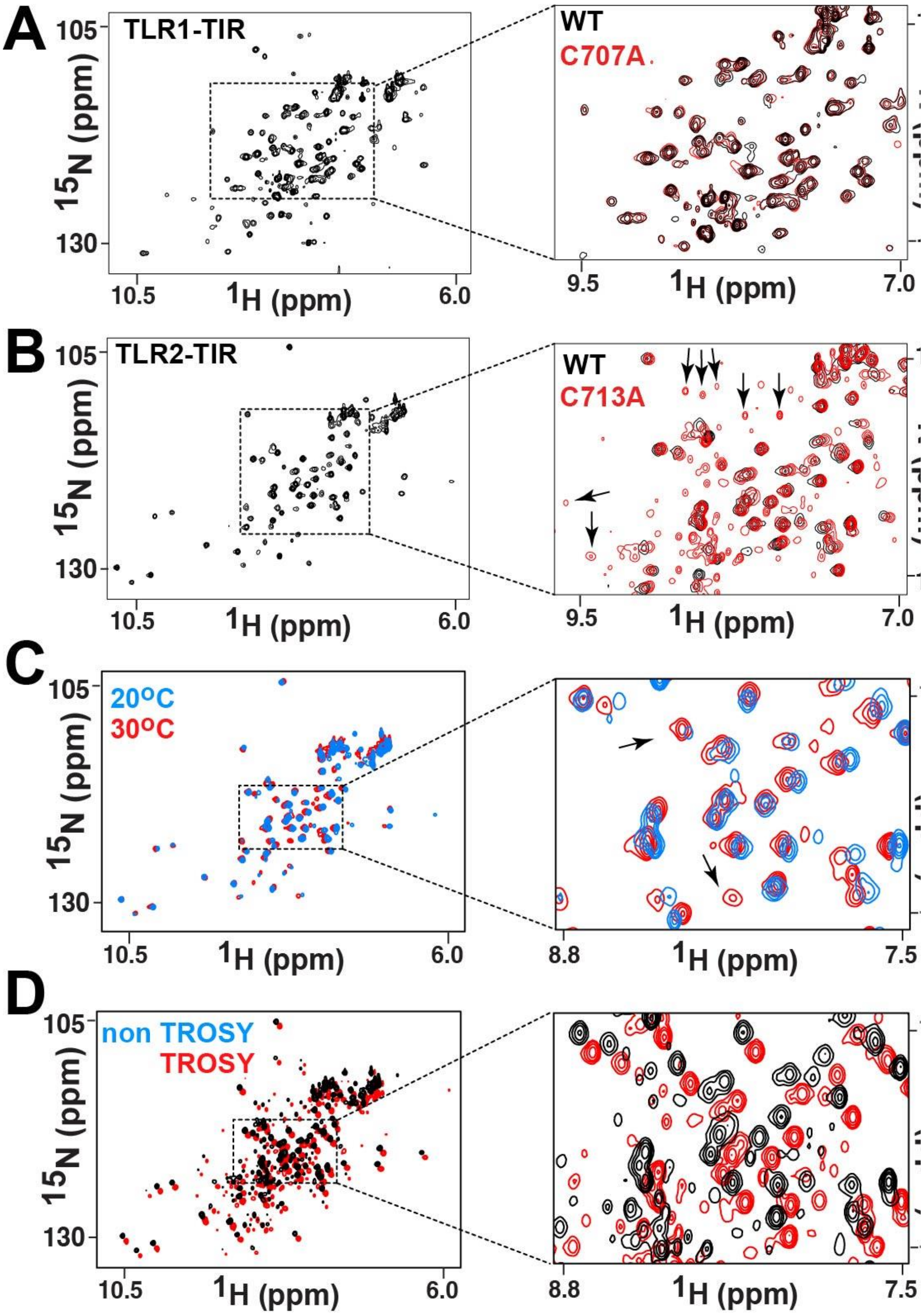

Figure S4. Spectral comparisons of TLR1-TIR and TLR2-TIR WT. A) ^{15}N-HSQC of TLR1-TIR WT (black) and the C707A mutant (red). B) ^{15}N-HSQC TLR2-TIR WT (black) and the C713A mutant (red). Arrows delineate new resonances that emerge within the mutation. C) ^{15}N-HSQC TLR2-TIR WT at 20 °C (blue) and 30 °C (red). Arrows delineate resonances that exhibit higher intensities at the higher temperature. D) ^{15}N-HSQC TLR2-TIR WT non-TROSY (black) and TROSY red).

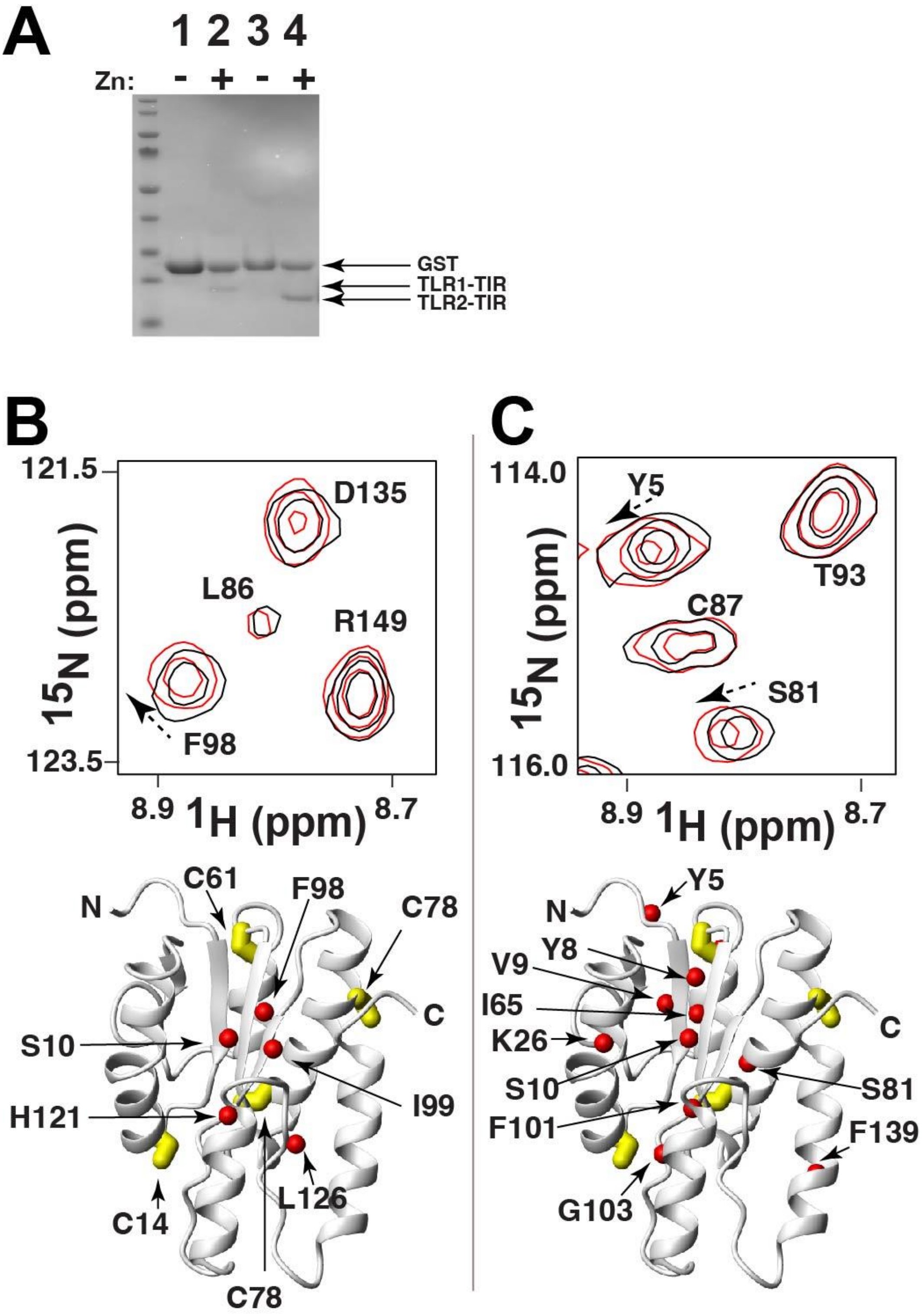

Figure S5. GST control pull-downs and minor spectral changes induced to IL-1R8 upon addition of TLR1-TIR and TLR2-TIR. A) GST control pull-downs in the absence and presence of 200 μM Zn for untagged TLR1-TIR (lanes 1, 2, respectively) and untagged TLR2-TIR (lanes 3, 4, respectively). B) ^{15}N-HSQC of IL-1R8-TIR alone (black) and in the presence of TLR1-TIR (red) with perturbed resonances mapped onto the low-resolution structure of IL-1R8-TIR (red balls). C) ^{15}N-HSQC of IL-1R8-TIR alone (black) and in the presence of TLR2-TIR with perturbed resonances mapped onto the low-resolution structure of IL-1R8-TIR (red balls).

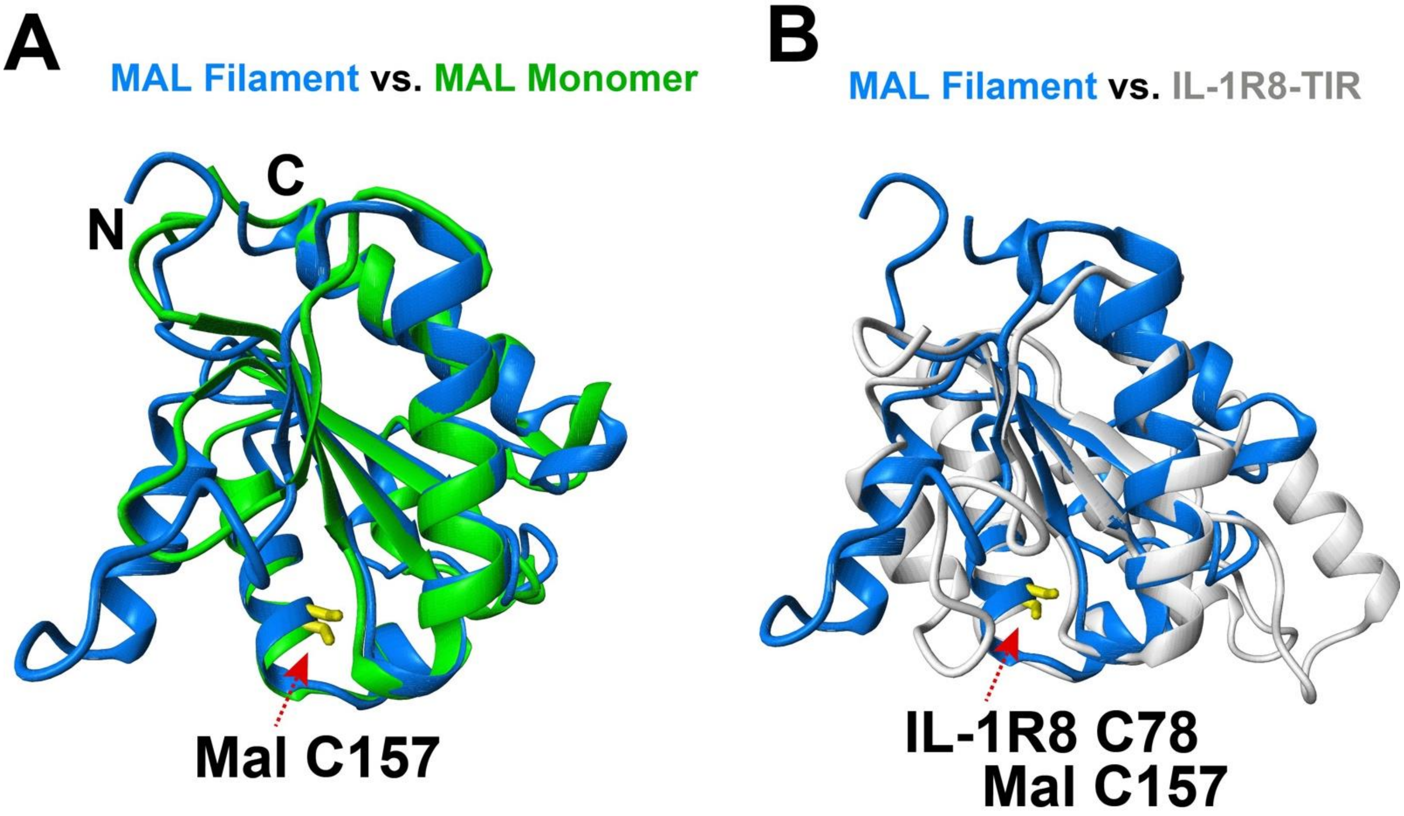

Figure S6. IL-1R8-TIR comparison to MAL. A) Least squares superposition of MAL alone (5UZB, green) and within filamentous MAL (5UZB, blue). The RMSD is 0.95 Å over 81 residues and illustrates the large conformational change that occurs in MAL polymerization. The arrow (red) delineates the C156 within the conserved "WC" of MAL (yellow). B) Least squares superposition of filamentous MAL (5UZB, blue) and the IL-1R8-TIR computed here (white). The arrow (red) delineates C156 and C789 within the conserved "WC" of MAL (yellow) and within IL-1R8-TIR (yellow).

Supplementary Tables

Table S1. Proteomic analysis of TLR1-TIR and TLR2-TIR disulfide exchange. Shown are the total number of peptide cross-links both prior to (Pre- **Table S1:** Proteomic analysis of TLR1-TIR and TLR2-TIR disulfide exchange. Shown are the total number of peptide cross-links both prior to (Pre-Treatment) and after (Post-Treatment) incubation with 5 mM TCEP).

Cross-link	Pre-Treatment	Post-Treatment
TLR1(C667)-TLR1(C686)	62	23
TLR2(C640)-TLR2(C640)	6	0
TLR2(C640)-TLR2(C673)	84	18
TLR2(C640)-TLR2(C713)	27	12
TLR2(C673)-TLR2(C713)	2	0
TLR2(C713)-TLR2(C713)	4	4
TLR1(C667)-TLR2(C640)	23	0
TLR1(C667)-TLR2(C673)	12	0
TLR1(C667)-TLR2(C713)	11	0
TLR1(C686)-TLR2(C640)	32	1
TLR1(C686)-TLR2(C673)	6	0

Table S2. Relaxation rates used for the determination of the TLR2-TIR correlation time.

#Peak (random numbering)	R2/R1
11	22.55 +/- 1.576
12	25.82 +/- 1.639
13	20.66 +/- 1.214
15	43.07 +/- 4.344
16	24.75 +/- 1.428
30	16.67 +/- 0.819
17	22.81 +/- 0.774
31	21.94 +/- 0.753
18	21.13 +/- 0.61
32	22.12 +/- 0.59
19	14.63 +/- 0.623
33	22.28 +/- 2.316
34	21.18 +/- 0.992
35	23.25 +/- 0.408
37	16.86 +/- 1.166
51	25.55 +/- 3.674
38	22.60 +/- 1.174
39	19.85 +/- 1.584
53	20.10 +/- 1.001
54	17.78 +/- 3.537
55	19.23 +/- 1.357
56	22.85 +/- 2.482
1	21.32 +/- 1.033
57	52.58 +/- 14.191
2	23.77 +/- 2.179
58	66.85 +/- 24.629
3	19.62 +/- 0.693
5	31.81 +/- 0.693

6	3.12 +/- 0.183
8	15.72 +/- 0.305
9	22.65 +/- 2.629
20	39.63 +/- 2.454
21	14.96 +/- 1.652
23	21.76 +/- 0.97
24	21.54 +/- 0.618
25	2.83 +/- 0.86
26	22.46 +/- 3.314
40	19.87 +/- 0.663
41	17.31 +/- 0.293
28	17.83 +/- 3.005
42	10.10 +/- 0.161
29	11.49 +/- 0.411
43	31.96 +/- 3.868
44	19.27 +/- 1.559
45	24.79 +/- 1.781
47	18.61 +/- 0.828
61	22.36 +/- 0.886
48	21.29 +/- 4.325
62	23.34 +/- 1.213
49	2.32 +/- 0.113
63	21.68 +/- 0.593

Average R2/R1:	22.18 +/- 1.48
Number of residues within this range:	42
Correlation time at 900:	10.03 ns

Table S3. Relaxation rates for the IL-1R8-TIR domain at 900 MHz.

Residue	R2 (Hz)	R1 (Hz)
2Gly	12.84478 +/- 0.15271	0.94533 +/- 0.01898
4Leu	20.38253 +/- 0.24311	0.63475 +/- 0.01330
5Tyr	27.65209 +/- 0.45253	0.53774 +/- 0.01886
6Asp	25.58306 +/- 1.44621	0.57964 +/- 0.02729
7Ala	26.81444 +/- 0.71994	0.56823 +/- 0.00726
8Tyr	26.30786 +/- 2.06287	0.57065 +/- 0.02117
9Val	27.35710 +/- 1.68606	0.49661 +/- 0.03451
10Ser	26.48628 +/- 0.83120	0.56903 +/- 0.02748
11Tyr	26.79455 +/- 2.03723	0.56469 +/- 0.05272
13Asp	20.00646 +/- 3.19935	
16Glu	30.10366 +/- 1.57070	0.58526 +/- 0.01608
17Asp	25.24896 +/- 0.45740	0.56889 +/- 0.01460
18Arg	26.34011 +/- 1.44318	0.56645 +/- 0.02416
20Phe	27.58418 +/- 0.24244	0.51444 +/- 0.01280
21Val	27.95330 +/- 1.19308	0.57015 +/- 0.02755
22Asn	26.87086 +/- 0.30486	0.53242 +/- 0.00593
23Phe	26.29377 +/- 1.19274	0.55052 +/- 0.01401
24Ile	24.75956 +/- 0.29934	0.53933 +/- 0.01020
25Leu	29.07835 +/- 0.60071	0.50293 +/- 0.01664
26Lys	27.87217 +/- 4.61496	
28Gln	24.98351 +/- 0.95385	0.54077 +/- 0.01300
29Leu	26.25407 +/- 0.40423	0.52640 +/- 0.00824
30Glu	27.93744 +/- 0.46659	0.57484 +/- 0.01392
32Arg	25.47611 +/- 0.56769	0.59674 +/- 0.00955
33Arg	26.41327 +/- 1.02885	0.59451 +/- 0.01228
34Gly	25.24151 +/- 0.51501	0.59566 +/- 0.00648
37Leu	23.71479 +/- 0.51588	0.50047 +/- 0.01693
38Phe	25.16337 +/- 0.35010	0.52314 +/- 0.01123
39Leu	25.61259 +/- 1.19716	0.46647 +/- 0.02173
40Asp	26.03631 +/- 0.93427	0.56455 +/- 0.02196
52Ala	49.01324 +/- 3.84825	0.71081 +/- 0.03310
53Asp	28.18382 +/- 0.66533	0.66101 +/- 0.00929
54Leu	28.43967 +/- 0.58425	0.60090 +/- 0.02419
59Ser	27.62894 +/- 0.54369	0.54979 +/- 0.03363
60Arg	28.59764 +/- 0.43699	0.57467 +/- 0.02423
61Cys	28.11059 +/- 0.76487	0.53871 +/- 0.02304
63Arg	24.68463 +/- 0.49510	0.54492 +/- 0.00880
64Leu	25.99678 +/- 0.83606	0.54686 +/- 0.04139
65Ile	24.42554 +/- 1.21069	0.58165 +/- 0.03770
66Val	25.33252 +/- 0.42361	0.50407 +/- 0.01996
67Val	23.20464 +/- 0.62337	0.53375 +/- 0.02650
69Ser	33.31733 +/- 3.42429	0.56194 +/- 0.05905
70Asp	28.05121 +/- 0.87355	0.57482 +/- 0.00839
71Ala	27.76962 +/- 0.62645	0.59965 +/- 0.02841
81Ser	35.85590 +/- 3.02165	0.55790 +/- 0.06687
82Phe	38.75259 +/- 2.80607	0.64516 +/- 0.10344
86Leu	39.36076 +/- 4.95660	0.67577 +/- 0.12380

87Cys	28.21927 +/- 1.43031	0.52905 +/- 0.03614
90Leu	29.93696 +/- 2.69355	0.52786 +/- 0.02839
91Glu	27.01901 +/- 0.45548	0.55184 +/- 0.01744
92Leu	26.85516 +/- 0.96087	0.57819 +/- 0.02620
93Thr	30.91952 +/- 0.89210	0.49752 +/- 0.01314
94Arg	23.26210 +/- 0.40882	0.57753 +/- 0.01861
95Arg	26.12885 +/- 1.95473	0.61427 +/- 0.04338
97Ile	25.68468 +/- 0.74149	0.47739 +/- 0.02646
98Phe	24.93840 +/- 0.38021	0.54218 +/- 0.01808
99Ile	24.43527 +/- 0.70250	0.52592 +/- 0.02447
100Thr	29.41040 +/- 2.64223	0.54609 +/- 0.05167
101Phe	36.77050 +/- 5.36972	0.53042 +/- 0.10206
102Glu	24.24858 +/- 1.24220	0.62655 +/- 0.02322
103Gly	38.90197 +/- 5.26587	0.62077 +/- 0.05448
106Arg	24.72000 +/-2.27658	0.81400 +/- 0.04422
107Asp	22.65218 +/- 0.50523	0.89863 +/- 0.02529
109Ala	26.57803 +/- 1.14597	0.71853 +/- 0.03401
110His	30.50750 +/- 4.73113	0.62848 +/- 0.05494
113Leu		0.62215 +/- 0.15689
115Leu	26.34182 +/- 0.47074	0.60494 +/- 0.01423
116Leu	25.38653 +/- 3.06669	0.62066 +/- 0.05802
119His	24.89288 +/- 1.02522	0.57522 +/- 0.02275
120Arg	27.62530 +/- 0.78111	0.61470 +/- 0.04518
121His	27.77026 +/- 0.43972	0.61364 +/- 0.03952
122Leu	28.65158 +/- 0.44697	0.56336 +/- 0.01194
123Val	29.79103 +/- 1.18505	0.58825 +/- 0.00982
124Thr	25.27219 +/- 0.32757	0.62180 +/- 0.02040
125Leu	23.35848 +/- 0.36998	0.52828 +/- 0.01741
126Leu	27.53045 +/- 0.57958	0.55262 +/- 0.01210
127Leu	22.84709 +/- 0.46034	0.56189 +/- 0.01315
128Trp	28.62688 +/- 2.55954	0.49543 +/- 0.02071
132Ser	28.81341 +/- 0.54331	0.64036 +/- 0.01039
133Val	31.01203 +/- 3.63508	0.74325 +/- 0.04149
134Thr	26.02992 +/- 0.41843	0.68253 +/- 0.01410
136Ser	30.70689 +/- 1.47509	0.70889 +/- 0.03718
137Ser	28.15995 +/- 0.09075	0.67946 +/- 0.01359
138Asp	25.82225 +/- 0.87835	0.65200 +/- 0.01282
139Phe	29.07258 +/- 0.43053	0.60632 +/- 0.01542
140Trp	28.57914 +/- 0.99241	0.55459 +/- 0.03516
141Lys	23.57688 +/- 3.04879	0.66641 +/- 0.09354
142Glu	28.37152 +/- 0.51339	0.55110 +/- 0.03723
143Val	27.57986 +/- 0.92028	0.57377 +/- 0.01496
146Ala	31.28818 +/- 0.49897	0.53698 +/- 0.00825
147Leu	27.26374 +/- 0.79028	0.55992 +/- 0.02161
149Arg	19.03508 +/- 0.37492	0.76519 +/- 0.00256
150Lys	13.36803 +/- 0.61245	1.01743 +/- 0.06416
